# Therapeutic efficacy of artemether-lumefantrine combination in the treatment of uncomplicated malaria among children under 5 years in 3 ecological zones in Ghana

**DOI:** 10.1186/1475-2875-11-S1-P107

**Published:** 2012-10-15

**Authors:** Benjamin Abuaku, Nancy Duah, Lydia Quaye, Neils Quashie, Kwadwo Koram

**Affiliations:** 1 Epidemiology Department, Noguchi Memorial Institute for Medical Research, College of Health Sciences, University of Ghana, P. O. Box LG581, Legon, Ghana

## Background

In 2008 artemether-lumefantrine and dihydroartemisinin-piperaquine were added to amodiaquine - artesunate as first line drugs for uncomplicated malaria in Ghana. The introduction of new drugs calls for continuous monitoring of these drugs to provide timely information on trends of their efficacy and safety to enhance timely evidence-based decision making by the National Malaria Control Programme. In this regard, we monitored the therapeutic efficacy of artemether - lumefantrine from September 2010 to April 2011 in 4 sentinel sites representing the 3 main ecological zones of the country.

## Materials and methods

The study population involved all children aged between 6 and 59 months presenting at the Out-Patient Department (OPD) of a study site clinic with symptoms suggestive of malaria. Using the 2009 WHO protocol for surveillance of antimalarial drug efficacy, primary outcomes for the study were treatment outcomes on Day 14 and Day 28 for the different ecological zones whilst secondary outcomes were patterns of fever and parasite clearance as well as gametocyte carriage and haematological responses. The Institutional Review Board of the Noguchi Memorial Institute for Medical Research, University of Ghana, reviewed and approved the study.

## Results

Per-protocol analysis showed that the overall pcr-corrected cure rates on day 14 and day 28 were 96.5% (95% CI: 92.1, 98.6) and 95.4% (95% CI: 90.3, 98.0), respectively, with statistically significant differences between the ecological zones. The 90.4% day-28 cure rate observed in the savannah zone (95% CI: 78.2, 96.4) was significantly the lowest compared with 100% (95% CI: 93.2, 99.9) in the forest zone and 93.8% (95% CI: 77.8, 98.9) in the coastal zone (*P*=0.017). Fever and parasite clearance were slower among children enrolled in the savannah zone. The proportion of children still febrile on day 1 post-treatment was significantly highest in the savannah zone (42.9%; 95% CI: 30.0, 56.7) compared with the forest zone (16.7%; 95% CI: 9.5, 27.2) and the coastal zone (10.5%; 95% CI: 3.4, 25.7) (*P*=0.000). Additionally, 14.5% (95% CI: 6.9, 27.2) of the children enrolled in the savannah zone were parasitaemic on day 2 post-treatment whilst no child was parasitaemic on the same day in the forest and coastal zones. Gametocytaemia after day 3 post-treatment was rare in all the zones. Mean haemoglobin concentration significantly increased only in the forest zone from 10.1g/dl (95% CI: 9.6, 10.5) on day 0 to 11.0g/dl (95% CI: 10.6, 11.4) on day 28 (*p*=0.004).

## Conclusions

We conclude that AL remains efficacious in Ghana with significant ecologic zonal differences. The savannah zone may be a potential zone for any emergence of resistant alleles as a result of the slower parasite clearance observed in the zone.

